# The Global Challenge of Antimicrobial Resistance: Mechanisms, Case Studies, and Mitigation Approaches

**DOI:** 10.1002/hsr2.71077

**Published:** 2025-07-23

**Authors:** Abubakar Nazir, Awais Nazir, Varisha Zuhair, Shafaq Aman, Safi Ur Rehman Sadiq, Abdul Haseeb Hasan, Maryam Tariq, Latif Ur Rehman, Mubarak Jolayemi Mustapha, Deusdedith Boniphace Bulimbe

**Affiliations:** ^1^ Department of Medicine King Edward Medical University Lahore Pakistan; ^2^ The Jewish Hospital‐Mercy Health Cincinnati Ohio USA; ^3^ Oli Health Magazine Organization, Research and Education Kigali Rwanda; ^4^ Jinnah Postgraduate Medical Centre Karachi Pakistan; ^5^ Karachi Medical and Dental College Karachi Pakistan; ^6^ Department of Allied Health Sciences Iqra National University Peshawar Pakistan; ^7^ Faculty of Basic Medical Sciences University of Ilorin Ilorin Nigeria; ^8^ School of Medicine and Dentistry The University of Dodoma Dodoma Tanzania

**Keywords:** antimicrobial resistance, bacterial adaptation, global threat, horizontal gene transfer, public health, resistance mechanisms

## Abstract

**Background and Aims:**

Antimicrobial resistance (AMR) is projected to cause 10 million deaths annually by 2050 if left unaddressed, posing a severe threat to global health and modern medicine. This review analyzes the molecular and ecological mechanisms underlying antibiotic resistance and evaluates global efforts aimed at containment to identify actionable strategies to mitigate AMR's escalating impact.

**Methods:**

A systematic literature review was performed using databases including PubMed, ScienceDirect, Scopus, Google Scholar, and Web of Science, focusing on peer‐reviewed studies from 2000 to 2024. Search terms included “antibiotic resistance,” “resistance mechanisms,” “horizontal gene transfer,” and “AMR epidemiology.” A total of 152 articles were selected based on predefined inclusion criteria relevant to resistance mechanisms, epidemiological data, clinical outcomes, and public health interventions.

**Results:**

Findings underscore three dominant resistance pathways: target site modification, enzymatic degradation (e.g., β‐lactamases), and horizontal gene transfer via plasmids and transposons. Notably, resistance to last‐resort antibiotics (e.g., colistin, carbapenems) is rising in pathogens such as *Klebsiella pneumoniae* and *Acinetobacter baumannii*, with treatment failure rates exceeding 50% in some regions. Surveillance gaps and unregulated antibiotic use, especially in LMICs, further accelerate resistance spread. Only a limited number of new antibiotic classes have been approved since 2010, underscoring the innovation gap.

**Conclusion:**

AMR is a quantifiable, escalating crisis that undermines decades of progress in infectious disease control. Tackling it requires coordinated action: strengthening antimicrobial stewardship, incentivizing antibiotic R&D, integrating environmental and clinical surveillance under One Health frameworks, and implementing global policy reforms. Without prompt action, AMR could surpass cancer in annual mortality by mid‐century.

## 1 Introduction

Antimicrobial resistance (AMR) is defined as the ability of microorganisms to survive and remain viable despite exposure to antimicrobial agents [1, 2]. Various types of antimicrobial agents, including antibiotics, disinfectants, and food preservatives, are used to combat microorganisms by reducing their growth, inhibiting their reproduction, or killing them outright. These agents can be natural, semi‐synthetic, or synthetic, each with distinct mechanisms of action [1, 2]. They can induce significant changes at the metabolic and physiological levels, such as altering cell wall synthesis (e.g., β‐lactams and glycopeptides), inhibiting protein synthesis (e.g., macrolides and tetracyclines), disrupting metabolic pathways (e.g., sulfonamides), or interfering with DNA replication and translation (e.g., fluoroquinolones) [3, 4, 5]. AMR is a significant public health issue in the 21st century and poses a serious threat to our ability to prevent and treat a variety of infections caused by bacteria, parasites, viruses, and fungi [6]. The first World Health Organization (WHO) global report on AMR surveillance revealed the worldwide extent of AMR and highlighted significant gaps in existing surveillance systems [6]. Drug‐resistant infections contributed to more than 4.95 million deaths globally in 2019, and without urgent intervention, this number could rise to 10 million deaths annually by 2050 [6, 7, 8]. Several recent outbreaks linked to AMR have underscored the severity of this crisis. The global spread of carbapenem‐resistant *Klebsiella pneumoniae* (CRKP) has been a major concern, particularly in healthcare settings, where it causes severe pneumonia, bloodstream infections, and urinary tract infections with limited treatment options [6]. In 2017, an outbreak of CRKP in Italy led to high mortality rates among hospitalized patients due to the pathogen's resistance to last‐line antibiotics [6, 9]. Similarly, methicillin‐resistant *Staphylococcus aureus* (MRSA) remains a leading cause of hospital‐acquired infections worldwide, with outbreaks occurring in intensive care units and postsurgical patients. In the United States, MRSA is responsible for an estimated 10,000 deaths annually [6, 10] (Table 1).

**Table 1:** Comprehensive table of antibiotics, their modes of action, and resistance mechanisms.

Class	Mechanism of action	Target pathogens	Common resistance
β‐lactams	Inhibit cell wall synthesis via PBPs	Broad (Gram+ and Gram−)	β‐lactamases, altered PBPs, porin loss, efflux
Glycopeptides	Bind D‐Ala‐D‐Ala to block peptidoglycan synthesis	Gram+ (MRSA, VRE)	D‐Ala‐D‐Lac modification (VanA)
Macrolides	Inhibit 50S ribosome – block protein elongation	Gram+, atypicals (Mycoplasma, Chlamydia)	Efflux (msrA), ribosome methylation (erm)
Tetracyclines	Inhibit 30S ribosome – block tRNA binding	Broad‐spectrum incl. intracellular	Efflux (tetA), ribosome protection (tetM)
Aminoglycosides	Bind 30S ribosome – cause mRNA misreading	Aerobic Gram−, some Gram+ (Enterococcus)	Modifying enzymes (aac, ant), 16S methylation
Fluoroquinolones	Inhibit DNA gyrase & topoisomerase IV	Broad‐spectrum	gyrA/parC mutations, efflux (qnr), plasmids
Sulfonamides	Inhibit folic acid synthesis via DHPS	Broad, some protozoa	dhps mutations, PABA overproduction, efflux
Oxazolidinones	Inhibit 50S – block ribosome assembly	Gram+ (MRSA, VRE)	23S rRNA mutation, cfr methylation
Polymyxins	Disrupt cell membrane (LPS interaction)	Gram− (Pseudomonas, Acinetobacter)	LPS modification (mcr‐1), efflux
Lincosamides	Inhibit 50S – block protein elongation	Gram+, anaerobes	erm‐mediated methylation, efflux
Rifamycins	Inhibit RNA polymerase – block transcription	TB, Neisseria, Staphylococcus	rpoB mutations, efflux
Fusidane	Inhibits EF‐G – halts protein elongation	Staphylococcus, Corynebacterium	fusA mutations, efflux (fusB)

Another alarming example is the emergence of drug‐resistant *Neisseria gonorrhoeae*, which has rendered first‐line treatments ineffective in many regions. In 2018, the United Kingdom reported its first case of a gonorrhea strain resistant to ceftriaxone and azithromycin, raising concerns about the potential for untreatable sexually transmitted infections [6, 11]. Additionally, multidrug‐resistant (MDR) *Salmonella* strains have led to outbreaks linked to contaminated food products, such as the extensively drug‐resistant (XDR) *Salmonella typhi* outbreak in Pakistan, which left thousands infected and was only treatable with a single remaining antibiotic [6, 12]. The emergence and rapid spread of AMR are driven by multiple interconnected factors, including the misuse and overuse of antibiotics in human medicine, veterinary practice, and agriculture. In many low‐ and middle‐income countries, the unrestricted availability of antibiotics, coupled with inadequate regulatory oversight, leads to self‐medication, inappropriate prescribing practices, and incomplete treatment regimens, major contributors to the selection and propagation of resistant bacterial strains. In the agricultural sector, antibiotics are extensively used not only for therapeutic purposes but also as growth promoters and prophylactic agents in livestock and aquaculture, fostering the emergence of resistant pathogens that can be transmitted to humans through direct contact, food consumption, and environmental exposure [13]. Additionally, environmental reservoirs play a crucial role in AMR dissemination, as pharmaceutical waste, untreated industrial effluents, and inadequately treated hospital sewage facilitate the persistence and horizontal gene transfer (HGT) of resistance determinants among bacterial populations. Poor sanitation, inadequate infection control measures, and global travel further exacerbate the problem by enabling the transboundary spread of resistant pathogens [14].

Distal drivers of AMR, including rapid urbanization, population growth, poor healthcare infrastructure, and ineffective policy implementation, have further exacerbated the crisis [15]. Additionally, bacterial resistance is fueled by genetic mutations, HGT, and selective pressure from antimicrobial use [4, 9]. These factors have made AMR one of the most pressing global health challenges, threatening modern medicine and increasing the risk of untreatable infections. Given the urgency of the situation, it is crucial to develop novel therapeutic strategies targeting AMR mechanisms. In this review, we aim to analyze the current understanding of AMR pathways and explore innovative approaches that could be leveraged to combat resistance effectively.

## 2 Traditional Mechanisms of Antibiotic Resistance

### 2.1 Overview of Classical Antibiotic Resistance Mechanisms

**Figure 1:**
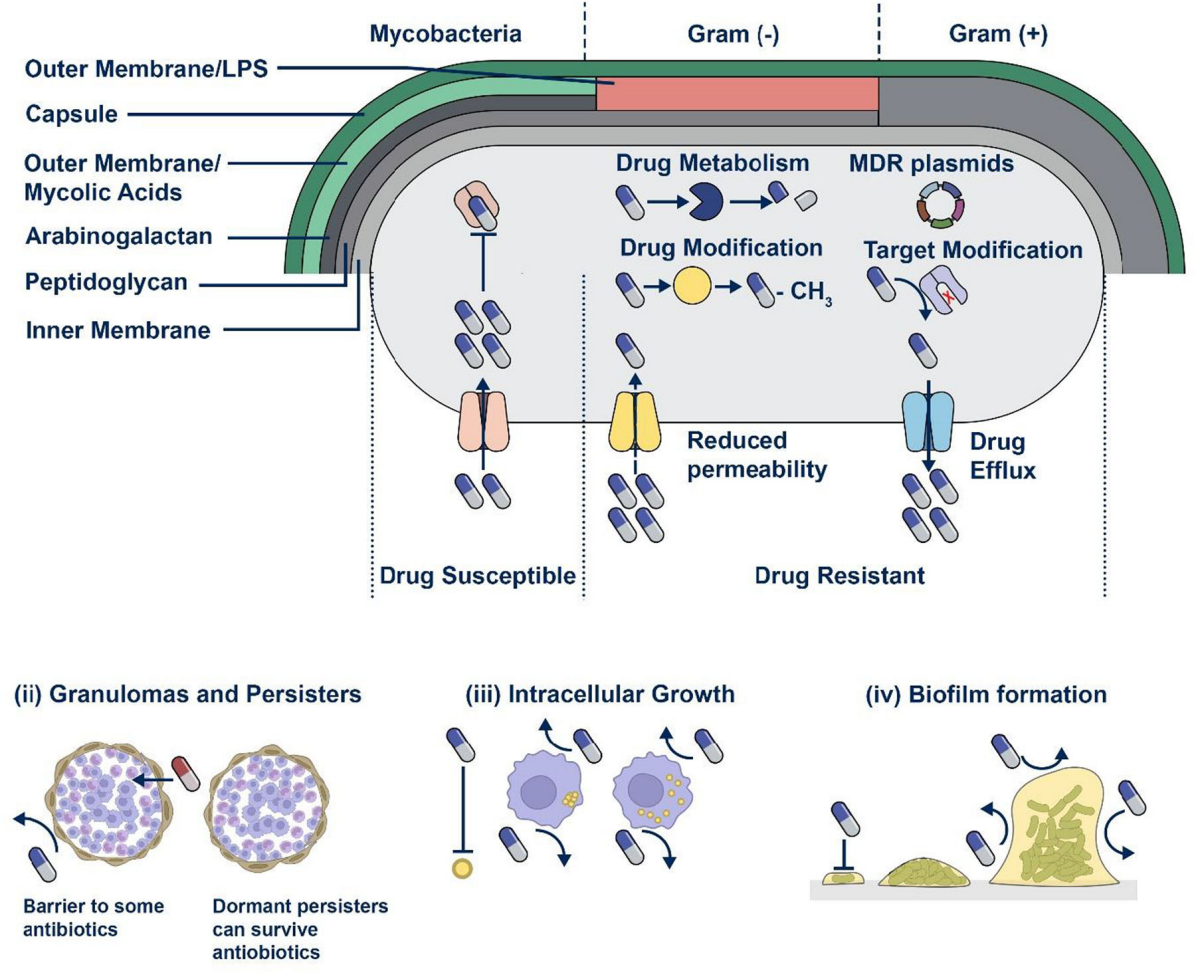
Structure of bacteria and classical antibiotic resistance mechanisms. It illustrates key bacterial resistance strategies, including efflux pumps (expelling antibiotics), enzymatic degradation (e.g., β‐lactamases), target modification (e.g., altered penicillin‐binding proteins in MRSA), reduced permeability (loss of porins in Gram‐negative bacteria), and metabolic bypass pathways. These mechanisms contribute to antimicrobial resistance, highlighting the need for innovative therapeutic strategies.

Antibiotic resistance in bacteria arises through several key mechanisms: alteration of drug targets, active efflux, reduced membrane permeability, and enzymatic inactivation of antimicrobial agents (Figure 1) [14]. These mechanisms, either individually or in combination, contribute to the growing threat of AMR. Clinically, the consequences of AMR are evident in the rise of drug‐resistant pathogens. MRSA, for instance, has become a major cause of hospital‐ and community‐acquired infections. Resistance is largely attributed to the mecA gene, which encodes PBP2a, an altered penicillin‐binding protein with low affinity for β‐lactams, rendering drugs like methicillin ineffective and complicating the management of pneumonia, sepsis, and osteomyelitis [5, 8, 15].

Likewise, extended‐spectrum β‐lactamase‐producing *Escherichia coli* and *K. pneumoniae* pose a major threat by hydrolyzing third‐generation cephalosporins, leaving carbapenems as one of the few effective treatments. Alarmingly, the emergence of carbapenem‐resistant Enterobacteriaceae, through genes such as *blaKPC*, *blaNDM*, and *blaOXA‐48*, has further restricted therapeutic options and is associated with high mortality, especially in bloodstream and ventilator‐associated infections [12, 14, 16].

MDR *Pseudomonas aeruginosa* represents another high‐risk pathogen, utilizing a combination of efflux pumps, porin mutations, and β‐lactamase production to evade treatment. It is particularly problematic in immunocompromised patients, including those with cystic fibrosis or burn injuries [12, 15].

These cases collectively underscore the global urgency of AMR. The evolution of resistance mechanisms continues to outpace drug development, threatening to render once‐effective antibiotics obsolete. Combating AMR requires a multipronged approach: investment in novel antimicrobials, enhanced global surveillance, and strict infection prevention and control strategies.

#### 2.1.1 Drug Inactivation by Enzymes

Antibiotics can be degraded by enzymes produced by bacteria, which also renders them ineffective as an antibacterial agent. As a result, the medicine fails to reach the bacterial cell and is destroyed [15]. Drug‐inactivating enzymes can be divided into three categories: β‐lactamases, aminoglycoside inactivating/modifying enzymes, and chloramphenicol acetyltransferases [9, 17]. The unstable hydrolytically sensitive chemical bonds in medicines can be targeted and broken down by the enzymes produced by both chromosomal and plasmid genes [18]. Many clinically relevant enzymes, such as blaCTX‐M, blaTEM, and blaSHV, and so forth, contain β‐lactamase enzymes that confer antibiotic resistance in bacteria [19].

#### 2.1.2 Alteration of Drug Target

The bacteria contain a large number of antibiotic‐binding targets [20, 21]. A key mechanism for drug resistance is the modification of the target site, which can make it challenging for antibiotics to adhere to the bacteria [18]. The two main hallmarks of this mechanism are witnessed in the resistance of drugs in Gram‐positive bacteria, as well as polymyxin. For instance, PBP2a, which is a low‐affinity binding protein, is created when *S. aureus*' PBP is transformed into it, leading to resistance against all β‐lactam antibiotics [22, 23].

#### 2.1.3 Modifications in the Permeability of the Outer Membrane

**Figure 2:**
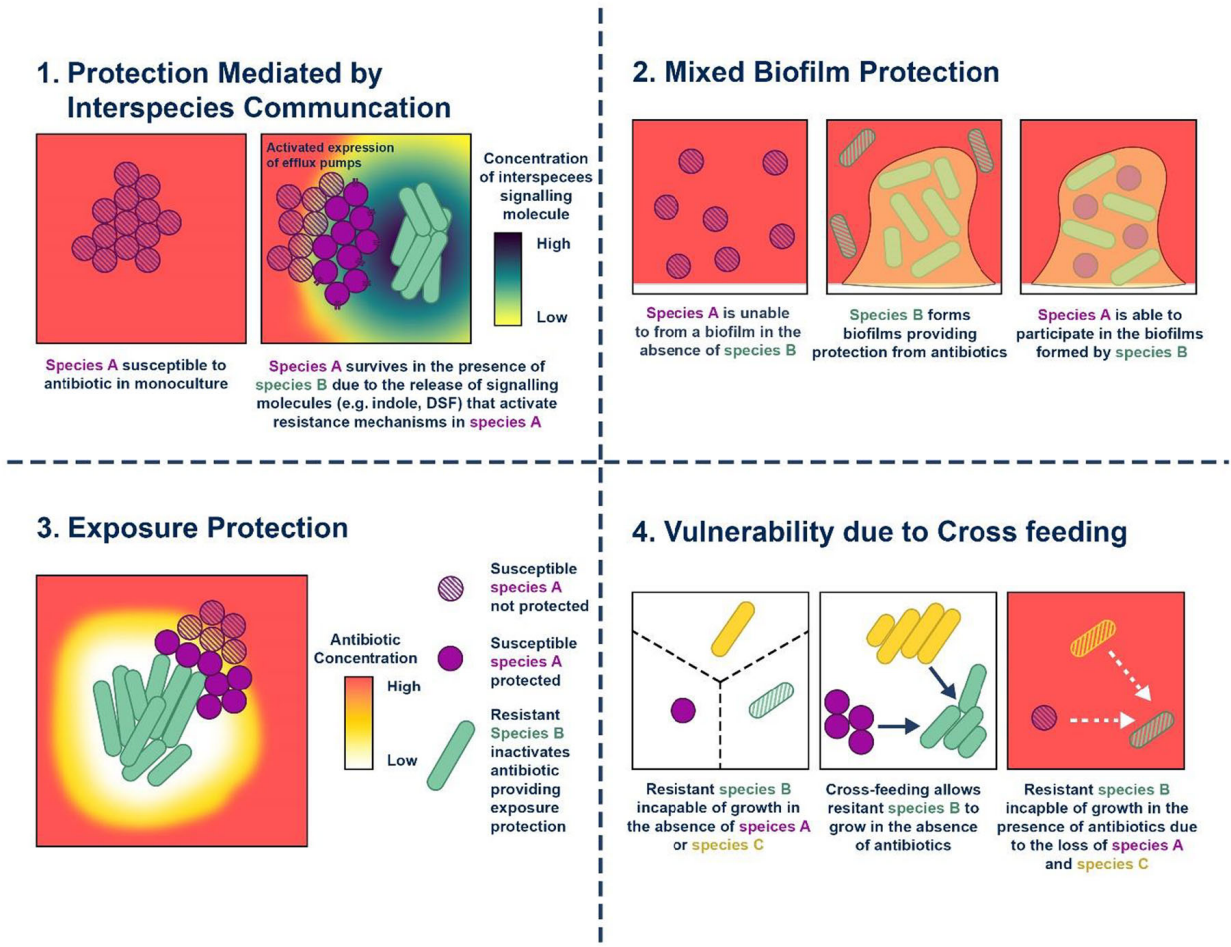
Mechanisms of antibiotic resistance. This figure illustrates key bacterial resistance strategies, including biofilm production that enhances bacterial protection by forming a physical barrier against antimicrobials, while exposure adaptation allows bacteria to survive sublethal antibiotic concentrations. Cross‐resistance mechanisms further contribute to multidrug resistance, increasing bacterial survival and limiting treatment options.

Beta‐lactam antibiotics mainly pass through hydrophilic channel proteins and penetrate the outer membrane of Gram‐negative bacteria, and mutations leading to changes in channel protein or a decrease in expression can lessen the susceptibility of the bacteria to multiple β‐lactam antibiotics. For instance, the absence of the channel protein named Opal D2 in hepatocytes results in imipenem resistance [22, 23]. The severe natural susceptibility of *P. aeruginosa* to antiseptics and antibiotics is due to its narrow outer membrane permeability [24]. It is anticipated that the mutation in the outer membrane porins gene may impact drug resistance (Figure 2).

#### 2.1.4 Active Antimicrobial Compound Efflux

It is often called a drug efflux system or an efflux pump mechanism. Antibiotic resistance occurs because the antibiotic's concentration inside the pathogenic bacteria is too low to have any antimicrobial activity. The process uses energy and interacts with numerous antibiotics [25]. Examples include the ability of the OprK protein in the outer membrane of *P. aeruginosa* to transport different antibiotics to the bacteria's surface [26]. Resistance nodulation division, major facilitator superfamily (MFS), multidrug toxic compound extrusion (MATE), small multidrug‐resistant (SMR), and ATP‐binding cassette (ABC) superfamilies are the most significant efflux transporters [27, 28, 29]. Several crucial genes are present in each efflux family, including acrB, emrE, bcr, cmr in MFS, mdtK, yeeO in MATE, ydgE in SMR, and macB in ABC [30, 31].

### 2.2 Examples of Well‐Known Antibiotic‐Resistant Pathogens and Their Resistance Mechanisms

#### 2.2.1 P. aeruginosa

*P. aeruginosa* is resistant to several antibiotics, including beta‐lactams, aminoglycosides, and quinolones [32, 33]. *P. aeruginosa* uses three main defense mechanisms to overcome antibiotic treatment: intrinsic resistance, adaptive resistance, or acquired resistance [33, 34]. This bacteria exhibits intrinsic resistance due to decreased outer membrane permeability, the formation of efflux pumps that remove medicines from the cell, and the creation of enzymes that inactivate antibiotics low outer membrane permeability, the development of efflux pumps that expel drugs from the cell and the synthesis of antibiotic‐inactivating enzymes are all components of *P. aeruginosa's* intrinsic resistance [32, 35]. Antibiotic resistance in *P. aeruginosa* may evolve by HGT or mutations [35]. *P. aeruginosa* acts by making biofilms in the lungs of infected patients [36]. These biofilms mimic as diffusion barriers and prevent antibiotics from penetrating bacterial cells [37].

#### 2.2.2 S. aureus

As MRSA infection and resistance rates increase, treatment with antibiotics is getting more and more difficult [38]. The majority of the intrinsic resistance mechanism is made up of three components, that is, outer membrane permeability, efflux pump, and excessive secretion of β‐lactamase enzymes [38]. A decrease in membrane permeability leads to *S. aureus* resistance to aminoglycosides, which in turn causes a reduction in drug consumption [39]. Multiple drug resistance in *S. aureus* is also influenced by its active mechanisms of drug efflux [40]. The cell membrane of *S. aureus* is home to three distinct types of proteins that pump multiple drugs outside, namely: QacA, NorA, and Smr [41, 42], of which QacA was thought to be a crucial component in MRSA by Noguchi et al. [43]. Another factor that contributes to resistant strains of *S. aureus*, the overproduction of β‐lactamases enzyme, which inhibits the impact of antibiotics via two processes [44]. First, the beta‐lactamase enzyme hydrolyzes and inactivates beta‐lactam drugs; followed by the “mechanism of pinching,” whereby a large quantity of beta‐lactamase binds rapidly and strongly to extracellular antibiotics, making them unable to penetrate the intracellular space and reach the target site, ultimately causing MRSA resistance to antibiotics [45, 46].

## 3 HGT: A Key Driver of Antibiotic Resistance Evolution

**Figure 3:**
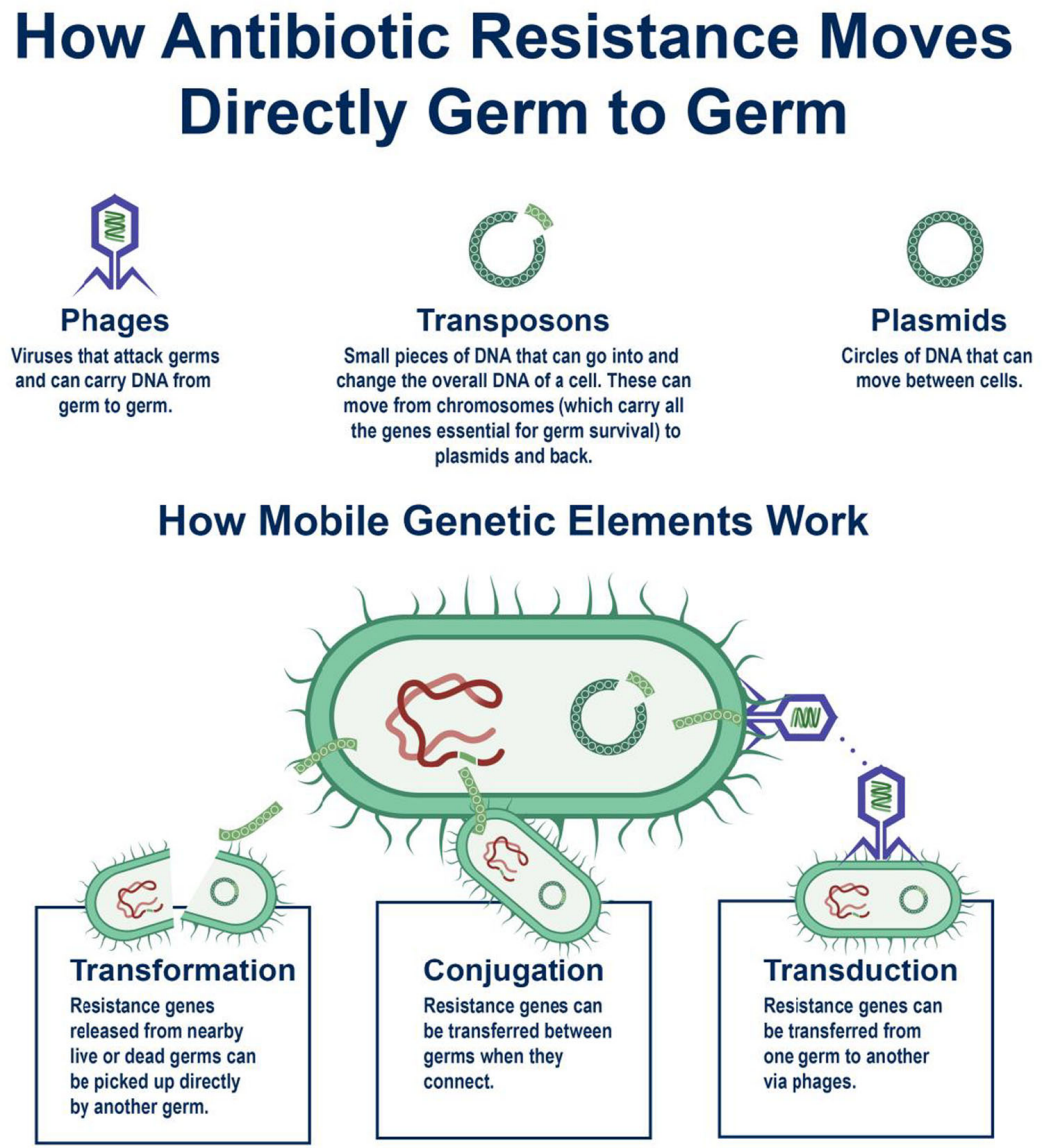
Spread of antibiotic resistance and genetic exchange. This figure illustrates how antibiotic resistance spreads through genetic exchange mechanisms, including horizontal gene transfer via transformation (uptake of free DNA), transduction (bacteriophage‐mediated transfer), and conjugation (plasmid exchange between bacteria). Mobile genetic elements such as transposons and integrons facilitate the dissemination of resistance genes. Environmental reservoirs, including wastewater, soil, and hospital settings, contribute to resistance gene propagation, accelerating the global spread of antimicrobial resistance.

Bacteria can develop antibiotic resistance in two different ways: either by spontaneous gene mutation or through HGT [47, 48]. The transmission of genetic information between organisms that are not related as parents and offspring is known as HGT [48]. The introduction of foreign DNA by the HGT mechanism is one of the factors responsible for the formation of antibiotic resistance in bacteria [48]. The majority of antibacterial substances employed in clinical settings are obtained from elements naturally occurring in the environment, primarily soil [49]. There is strong evidence suggesting that the emergence of antibiotic resistance genes (ARGs) in therapeutically important bacteria mostly originates from the environmental resistome [50]. Since bacteria that share the environment with these compounds carry internal genetic determinants of resistance. It has also been proven that the spread of antibiotic resistance is a result of this genetic exchange (Figure 3).

There have been three main ways for bacteria to take in foreign genetic material: the incorporation of bare DNA from the surrounding environment by a mechanism called transformation, transduction, which is mediated by viruses, and conjugation, in which DNA is transferred between bacteria through a conjugating pilus [48]. The most common type of HGT mechanism is transformation [48]. Conjugation is also a very efficient form of gene transfer that involves cell‐to‐cell contact and is frequently implicated in the development of resistance in hospital settings [51]. It has been discovered that contact with minimal limiting concentrations of two antibiotics, such as gentamycin and chloramphenicol, or aminoglycosides such as streptomycin stimulates conjugation between certain Gram‐negative bacteria [52] (Figure 3).

### 3.1 Role of Environmental Factors in Gene Transfer and Its Implications for AMR

The environment acts not merely as a passive backdrop but as an active conduit in the transmission and evolution of AMR. Gene exchange among microbial communities in soil, water bodies, and wastewater treatment plants drives the horizontal transfer of resistance traits, often mirroring or even preceding trends observed in clinical settings. Antibiotic residues from agricultural runoff and effluents from livestock operations introduce selective pressure in ecosystems, fostering conditions where resistance genes such as *blaNDM‐1* and *mcr‐1* emerge and proliferate beyond hospital walls [53, 54, 55]. Inadequately treated pharmaceutical waste amplifies this risk, transforming aquatic systems into genetic hotspots where resistance traits can cross species and ecological boundaries (Figure 3).

Urbanization and poor sanitation, particularly in low‐ and middle‐income countries, create densely populated microenvironments where resistant pathogens circulate between humans, animals, and the environment. These conditions erode the traditional barriers separating clinical and environmental reservoirs, complicating efforts to trace the origin or limit the spread of resistance. Moreover, climate change adds a poorly understood layer of complexity, altering microbial community dynamics, expanding the geographical range of pathogens, and increasing the persistence of resistance genes in aquatic and terrestrial environments.

Despite growing recognition of these interconnected pathways, current AMR surveillance efforts remain predominantly clinical, overlooking environmental reservoirs where early warning signs often manifest. This disconnect represents a significant gap in global AMR containment strategies. Addressing it demands not only tighter controls on waste management from the pharmaceutical and agricultural sectors but also the integration of environmental data into AMR surveillance frameworks. Environmental sampling, genomic monitoring of resistance genes, and cross‐sectoral data sharing under the One Health paradigm are critical to moving from reactive responses to proactive containment.

### 3.2 Plasmids, Transposons, and Integrons: Vehicles for HGT

Plasmids are small molecules of double‐stranded, spiral‐shaped DNA, whereas transposons are small DNA fragments that encode enzymes that move them from one DNA location to another, either on the same molecule of DNA or on a different molecule [30]. Plasmids and transposons are the two most significant mobile genetic elements (MGEs) because they both significantly contribute to the emergence and spread of antibiotic resistance by aiding in the conjugation process [53, 54].

Integrons play a crucial role in bacterial evolution by facilitating genetic exchange and promoting the acquisition of new resistance traits. They function by providing the necessary machinery for the integration and expression of newly acquired genes, thereby enhancing bacterial adaptability [13]. Transposons, MGEs responsible for gene transfer, exist in three distinct types but share common structural features, including inverted repeat sequences (25–200 bp) at their termini and a transposase enzyme with low site specificity. Many transposons carry single or multiple ARGs, such as tetracycline resistance (Tn10), ampicillin resistance (Tn3), or chloramphenicol resistance (Tn9) [55, 56]. In some cases, transposons and insertion sequences can combine to form complex genetic structures, such as Tn21, which may have originated from the integration of a transposable integron (In2) into a mercury‐resistant transposon (Tn501) [56]. Integrons further enhance bacterial adaptability by capturing and incorporating short gene cassettes at a specific integration site (attL), where an integrase enzyme (intI) facilitates site‐specific recombination, ensuring the efficient spread of AMR genes [57].

A broad study of the genes linked with attC in soil has identified them as anonymous genes, housekeeping genes, and ARGs, despite the fact that the majority of the investigated integron instances carry ARG cassettes [58, 59]. Although it is unknown how the different attCs migrate, the fact that many different forms of bacteria (both Gram‐positive and negative) contributed to the genes linked to sequences that are similar to the attC sequence in soil suggests that many bacteria have them [52, 60].

### 3.3 Case Studies of HGT‐Mediated Resistance Development in Different Bacterial Species

The effects of HGT events in clinical settings are further explained by infectious disease epidemics in the community that are caused by HGT. The spread of infections that were previously uncommon was facilitated by the dispersal of a plasmid encoding resistance to azithromycin, since conventional antibiotics were no longer effective. The independent acquisition of the same plasmid has led to multiple outbreaks involving different bacterial strains [52]. Immunocompromised patients, who have increased exposure to clinical environments, face a significantly higher risk of severe infections and mortality due to antibiotic‐resistant bacteria [52]. Plasmid transfer also affects the clinical resistance of Gram‐positive bacteria such as *S. aureus*. Conventional antibiotics have difficulty treating infections caused by MRSA, a common source of hospital‐acquired illnesses [52]. MRSA and other infections with resistance to other antibiotics are treated as a last resort with vancomycin. However, by horizontally acquiring a plasmid from *Enterococcus faecalis*, MRSA evolved into vancomycin‐resistant *S. aureus* (VRSA) [52].

## 4 Adaptive Responses and Phenotypic Heterogeneity

Phenotypic heterogeneity refers to the phenomenon in which genetically identical bacteria residing in a homogenous environment exhibit distinct traits, thus enabling the emergence of novel functional capabilities [61]. Even genetically identical bacterial cells can exhibit distinct behaviors and responses to stimuli [62]. The origin of heterogeneity in bacterial populations can be attributed to the stochastic nature of gene expression, which brings forth phenotypic variations in the generations [62]. Fundamentally, gene expression entails inherently stochastic biochemical reactions responsible for generating mRNA and proteins, ultimately leading to diversification [62]. Bacteria have adapted to leverage this phenomenon to their benefit by utilizing feedback‐driven circuits and forming increasingly diverse phenotypic subgroups [63]. It is hypothesized that individual cells undergo stochastic transitions between activating and deactivating a metabolic pathway, potentially providing a fitness advantage to a subpopulation [62]. In situations where a bacterial population faces sudden environmental changes, any subpopulations possessing advantageous traits that enable them to endure stressors like exposure to antibiotics will still have the opportunity to thrive, consequently enhancing the overall survival of the population [64].

Research has shown that subpopulations within genetically identical populations can have different antibiotic susceptibilities, as seen with persister cells [65]. Persisters are subsets of a bacterial population that can withstand antibiotic treatment in otherwise antibiotic‐sensitive cultures. They typically exhibit slow growth or growth suspension and can restart their growth after the subsidence of a severe stressor [66]. The existence of persister cells can lead to the resilience and resurgence of persistent bacterial infections and the development of antibiotic resistance during treatment [66]. The development of tolerance in persisters appears to operate through a mechanism involving the shutdown of their molecular targets. Bactericidal drugs exert their lethal effects by disrupting these targets rather than merely inhibiting them. Inactivating these targets serves as a defense mechanism, allowing persister cells to evade the lethal effects of antibiotics [67].

The gene expression profile of persisters was found to encompass toxin‐antitoxin modules along with other genes capable of impeding crucial cellular processes, particularly translation [68]. Notably, a marked escalation in the population of persisters was observed upon the overproduction of the RelE toxin, which acts as a translation inhibitor [68]. The inhibition of translation results in the cessation of vital cellular functions, thereby thwarting the action of antibiotics in corrupting their intended targets. This, in turn, contributes to the emergence of persister cells that exhibit multidrug tolerance [69]. Persister cells have been detected across all significant pathogens and are a leading factor behind chronic infections [69].

Other than the natural existence of persister cells within bacterial populations, antibiotic‐tolerant and resistant bacterial populations can also arise from specific external triggers. Bacteria are significantly influenced by environmental stress. It triggers numerous adaptive and protective reactions that modify gene expression patterns and cell physiology, ultimately affecting their susceptibility to antimicrobial agents [70]. This influence occurs through indirect mechanisms, such as stress‐induced growth cessation or dormancy, which render bacteria less vulnerable to antimicrobials that target actively growing cells. Additionally, stress can directly contribute to AMR by altering antimicrobial targets, mitigating changing membrane barrier functions, inducing resistance mutations, or encouraging resistant growth patterns like biofilms [70]. Among other stressors like nutrient starvation, hypoxia, pH fluctuations, changes in osmotic pressure, and extreme temperature shifts, one of the main stressors for bacteria is antibiotic exposure. Exposure to bactericidal agents can initiate protective responses in bacteria, potentially fostering resistance‐promoting reactions [70].

Antibiotics‐induced stress can activate the SOS (Save Our Souls) response in bacteria through DNA damage or replication arrest. For example, fluoroquinolone blocks DNA gyrase and trimethoprim impacts purine and pyrimidine synthesis [71, 72]. These effects lead to abnormal amounts of single‐stranded DNA within the cells, which is the primary signal for the SOS response. During the SOS response, bacteria can reorganize and alter their genetic makeup, leading to various changes in their characteristics. This response can result in the differential regulation of genes, leading to outcomes like increased virulence, enhanced persistence, and the development of antibiotic resistance [73]. SOS response also increases the mutation rate, called adaptive mutagenesis [73]. The higher mutation rate increases the chances of mutations that allow adaptation to the stressor. For instance, resistance to ciprofloxacin and rifampicin is due to mutations caused during the induction of the SOS response through the action of the error‐prone polymerases [74].

Notably, the SOS response can also be activated by minimal amounts of antibiotics, referred to as sub‐MIC (sub‐minimal inhibitory concentration). This holds significance because it implies that even the small quantities of antibiotics frequently detected in the environment, wastewater treatment facilities, hospitals, and soil can play a role in developing and disseminating antibiotic resistance [73]. Sub‐MICs can result in changes in gene expression, including genes related to resistance to oxidative stress, motility, virulence, and biofilm formation [73, 75]. Additionally, sub‐MICs of antibiotics increase mutation frequencies and promote the spread of ARGs even without direct DNA damage, potentially through the generation of reactive oxygen species that damage DNA and trigger the SOS response [73, 75].

## 5 Antibiotic Resistance in Biofilms

Biofilms are structured communities of bacteria encased in a self‐produced matrix of polysaccharides, proteins, and extracellular DNA. This matrix offers physical protection and biochemical resistance, shielding bacteria from environmental stressors such as UV radiation, extreme pH or salinity, nutrient scarcity, and—most notably—antibiotics and immune defenses [76].

Biofilm formation occurs on both artificial surfaces (e.g., catheters, stents, prosthetic joints) and natural tissues (e.g., teeth, heart valves, lungs in cystic fibrosis, chronic wounds, and the middle ear), contributing significantly to persistent and chronic infections [76]. The initial stage involves reversible attachment of planktonic (free‐floating) bacteria to a surface, aided by structures like flagella in motile species such as *P. aeruginosa* [77]. If not interrupted, this progresses to irreversible attachment, microcolony formation, and development of a mature biofilm—often reaching thicknesses of up to 50 μm and forming complex, mushroom‐like structures [78]. During early formation, bacteria remain more susceptible to antibiotics, underscoring the importance of timely prophylaxis [78].

The resistance exhibited by mature biofilms can be 10–1000 times greater than that of their planktonic counterparts [79]. This is due to several overlapping factors:

- **Physical barriers** within the matrix limit antibiotic diffusion.
- **Altered metabolic states**, especially in deeper layers with lower oxygen and nutrient levels, reduce antibiotic efficacy.
- **Adaptive gene expression** results in phenotypic changes, including upregulation of enzymes like β‐lactamases that degrade antibiotics [80, 81, 82, 83].

Several *strategies have been developed to overcome biofilm‐associated resistance*. Clinical use of *combination antibiotic therapy* remains a mainstay, especially in pulmonary *P. aeruginosa* infections, to enhance efficacy and reduce resistance emergence [84]. However, such approaches are not always sufficient, prompting the development of *novel anti‐biofilm strategies*, including:

- **Quorum sensing inhibitors**: Compounds such as halogenated furanones disrupt bacterial communication systems (e.g., N‐acyl homoserine lactones), preventing coordinated biofilm formation and virulence expression, particularly in Gram‐negative bacteria [85].
- **Surface modification**: Silver‐coated medical devices and anti‐adhesion agents (e.g., pilicides and curlicides) can inhibit initial bacterial attachment [86, 87].
- **Biofilm dispersal agents**: Signals like nitric oxide can trigger the dispersal of mature biofilms, rendering bacteria more susceptible to antibiotics [88].
- **Matrix‐disrupting enzymes**: DNase and other enzymes degrade extracellular polymers, allowing better antibiotic penetration [88].
- **Antimicrobial peptides (AMPs)**: Cationic amphipathic peptides, such as peptide 1018, not only kill bacteria but also disrupt quorum sensing and prevent biofilm formation by inhibiting the stringent stress response mediator (p)ppGpp [89, 90, 91, 92].

Lastly, *bacteriophages* offer a promising biological approach by targeting bacteria within biofilms. These viruses degrade the biofilm matrix, promote bacterial lysis, and improve antibiotic penetration. However, concerns remain about potential immune responses and phage resistance, requiring further research [93].

## 6 Antibiotic Resistance in the Microbiome

### 6.1 The Role of the Human Microbiome in Antibiotic Resistance Dissemination

Resistome composition, which includes all ARGs in the gut microbiome, is crucial for predicting how an individual's microbiome responds to antibiotics [94]. This is impacted by the emergence of antibiotic‐resistant pathogens and microbiome dynamics in which commensal microbes serve as a reservoir of ARGs in the human microbiome [94], which is influenced by direct and indirect factors; whereas direct factors are antibiotic usage, which applies at agriculture and clinic settings also being exposed to antimicrobials in hospital facilities [95, 96, 97]. Indirect factors comprise a variety of environmental stimuli, host genetics, and diet. Frequency of ARGs transfer depends on factors like MGEs, recipient and donor microbes' taxonomic similarity, environmental conditions, and distribution of ARGs in commensal microbes [94, 97, 98]. Again, the likelihood of ARGs transfer is increased by antibiotic use via selection in commensal strains [97].

### 6.2 Antibiotic‐Induced Dysbiosis and Its Impact on Resistance Development

Antibiotic‐induced dysbiosis involves a reduction in diversity of gut microbiota, alterations in the abundance of certain taxa, alterations in gene expression, protein activity, and gut metabolome, compromised colonization resistance to invading harmful bacteria, and emergence of ARGs due to administration of antibiotics [99, 100, 101]. There is a significant impact of antibiotic‐induced dysbiosis, such as reduction in diversity of gut microbiota, weakening the gut's ability to resist harmful bacteria, increasing susceptibility to infections due to compromised colonization resistance [102, 103]. Prolonged antibiotic use can result in short‐term consequences like *Clostridiodes difficile*‐associated diarrhea, *Helicobacter pylori* infections, and long‐term consequences like obesity, allergies, asthma, and inflammatory bowel disease [99, 104]. Vulnerability of specific populations, such as neonates and children, to the negative effects of antibiotic‐induced dysbiosis [105]. Neonates exposed to antibiotics early in life may experience long‐term impacts on their gut microbiota, which can lead to various health consequences, including an increased risk of childhood obesity, allergies, and asthma [102, 106].

### 6.3 Strategies to Restore and Maintain a Healthy Microbiome to Prevent Resistance

The use of probiotics to counteract the effects of antibiotic‐induced dysbiosis is a promising approach, and several studies support the efficacy in preventing or alleviating antibiotic‐associated dysbiosis and related health issues [102, 107, 108]. Prioritizing more emphasize on the importance of conducting more extensive further studies to isolate and sequence bacteriophages to better understand their role in the dissemination of ARGs [109, 110]. Such research will be crucial for developing strategies to address AMR effectively [109].

## 7 Role of MGEs in Antibiotic Resistance Dissemination

### 7.1 Integrating Genomic Islands and Resistance Cassettes Into the Bacterial Genome

A study was done in Korea, which paved light on how ARGs were acquired and disseminated in *Salmonella enterica serovar Gallinarum* (*S. Gallinarum*) strains [111]. Antibiotic resistance is controlled by multiple genes encoded by MGEs called gene cassettes, which can move in and out of specific receptor sites mediated by specific recombinase [112]. Integrons such as ln2 encode gene cassettes conferring resistance to streptomycin/spectinomycin, hence dissemination of antibiotic resistance, which has been observed to be aided by Tn21 transposon [113]. Increased use of antimicrobial drugs in poultry farms has increased antibiotic resistance in *S. Gallinarum* strains, especially in ciprofloxacin and gentamicin [114]. Comparison between two strains of *S. Gallinarum* isolated from infected chickens was compared with their whole genome sequence strains in this study, which showed that all strains harbor identical DNA carrying ARG cassettes but have variable resistance profiles due to differences in expression of specific resistance genes [111]. Promoters were identified to be responsible for the expression of ARGs and demonstrated how differences in promoter activity can lead to variations in resistance phenotypes [111].

### 7.2 Role of Bacteriophages in Transferring Resistance Genes

Bacteriophages are viruses that infect bacteria and are shown to be important carriers for the transmission of ARGs in the environment by the HGT mechanism, in which bacteriophages transfer ARGs in soil between different bacterial species, genera, or even phyla [110, 115]. This transfer can occur via transduction, a process where phages carry bacterial DNA, including ARGs, from one bacterium to another [116]. Also, bacteriophages have a surprisingly high frequency of gene transfer, especially when compared to other HGT mechanisms like conjugation [117]. This suggests that they play a significant role in the spread of antibiotic resistance. On top of that, bacteriophages do not require direct cell‐to‐cell contact for gene transfer, unlike some other mechanisms like conjugation [115, 116]. This means they can mediate gene transfer over longer distances and in various environmental conditions, and bacteriophages are highly stable and can withstand harsh environmental conditions, making them effective carriers of ARGs over a long time [116, 118]. Lastly, the transfer of ARGs via phages can be influenced by factors such as antibiotics, physicochemical characteristics of soil, bacterial community composition, and even nanomaterials. For example, antibiotics can enhance the ability of phages to transmit resistance [116, 119, 120, 121] (Table 2).

**Table 2:** Source of spread of antibiotic resistance across bacterial populations.

Major sources	Type and nature of potential environmental releases
Effluent and waste from healthcare facilities	Resistant microbes (including those with more abundant and diverse ARGs) and antimicrobial residues (particularly antimicrobial compounds of last resort) in hospital wastewater/effluent Antimicrobial products and residues in hospital solid wastes
Poor sanitation, sewage, and waste effluent	Releases from unused drugs disposed of in toilets, bins, or waste dumps Preventable use of antimicrobials due to disease burden caused by poor WASH conditions Urban runoff Effluent from septic tanks and wastewater treatment plants Fecal sludge and wastewater biosolids Leaching from open waste dumps Lack of sanitation or poorly functioning sanitation or fragmented systems (e.g., open defaecation, poorly contained pit latrines, septic tanks, and sewers) that contaminate water sources and spread AMR
Releases, effluent, and waste in animal production	Improper disposal of unused drugs Application of antibiotics and parasiticides in aquaculture that go directly into the environment Manure and effluent from aquatic and terrestrial animal production that may contain pharmaceutical residues, ARGs, and resistant microbes
Use of antimicrobials and manure in crop production	Inappropriate disposal of unused antimicrobials (e.g., fungicides) Untreated manure and wastewater that may contain pharmaceutical residues, ARGs, and resistant microbes intentionally applied to soil and crops Fungicides, herbicides, heavy metals, and antibiotics used in the production of food, feed, and raw materials
Effluent and waste from pharmaceutical manufacturing	Residual antimicrobials in solid wastes discharged from pharmaceutical fermentation processes High concentrations of antimicrobials in untreated effluent Resistant microbes in effluent if biological treatment is applied

### 7.3 Implications for the Spread of Antibiotic Resistance Across Bacterial Populations

ARGs in the environment, including soil, can contribute to the antibiotic resistance problem [116]. Soil acts as a reservoir for ARGs, which can eventually enter the food chain and pose a threat to human health [122]. Phages are robust entities in the environment, resistant to various stressors, and can persist over time [115, 123]. This widespread use of antibiotics in animal agriculture contributes to the selective pressure on bacteria and may lead to the development of antibiotic resistance due to significant use of antibiotics in poultry production, not only for treating infections but also as metaphylactic agents and growth promoters [109, 124, 125] (Table 2).

## 8 Evolutionary Trade‐Offs: Fitness Costs and Compensatory Mutations

The fitness cost of AMR refers to the trade‐off between a bacterium's ability to grow and compete in the absence of antibiotics versus its ability to survive in the presence of antimicrobial agents. Studies have shown that the fitness cost associated with resistance varies depending on the specific mutation, with some mutations imposing a significant burden on bacterial growth, while others have minimal impact. The extent of this cost is largely mutation‐dependent, as different resistance mechanisms affect bacterial physiology in diverse ways [126]. Detailed reconstructions of resistance mutations have revealed that in clinical settings, resistant strains often carry mutations that impose lower fitness costs, as determined by in vitro assays. This suggests that bacteria may preferentially retain mutations that balance resistance with viability [127]. In some cases, certain resistance mutations appear to have no detectable fitness cost, although it remains unclear whether these mutations carry subtle disadvantages that current methods fail to detect or if specific environmental conditions may reveal their effects.

One documented example is the K42R mutation in the 30S ribosomal protein S12, which confers streptomycin resistance in *E. coli*, *Mycobacterium tuberculosis*, and *Salmonella enterica* serovar Typhimurium without an apparent reduction in fitness [128, 129]. Interestingly, some studies have even suggested that certain resistance mutations can enhance bacterial fitness under specific conditions. Furthermore, the impact of resistance mutations can vary depending on the genetic background of the bacterial strain or clonal lineage, highlighting the role of epistatic interactions, where genetic factors influence the fitness effects of resistance mutations. Understanding these dynamics is crucial for predicting the evolutionary trajectory of resistant pathogens and designing effective strategies to mitigate AMR [130, 131, 132].

In fact, a meta‐analysis research revealed that certain resistance mutations in *Borrelia burgdorferi* and *Enterococcus faecium* exhibited an average fitness score > 1 without the use of antibiotics, whereas the mutations in organisms like *M. tuberculosis* and *S. aureus* were costly [133]. In addition, the GyrA C257T mutation, which provides quinolone resistance, was demonstrated to boost the performance of a single strain of Campylobacter in a chicken model with infections and reduce fitness once transferred into a different strain of the same bacterium, confirming how variable fitness costs may be the justification for epistatic consequences [134].

It is crucial to keep in mind that secondary mutations are one of the most common methods used to offset fitness costs. By altering the resistant allele of the altered protein's impacted function, removing the necessity for this process, or restoring the compromised protein's activity, mutations like these can improve microbial fitness [135]. Based on whether mutations happen at a single locus as the resistant mutation or at different loci, compensatory mutations may be classified as intragenic or extragenic [136]. Whereas the secondary mutations, primarily in the context of intragenic compensation, occasionally return the vulnerability to antimicrobial agents, it often happens that the compensated mutants retain their ANR [127] (Table 3).

**Table 3:** Current and emerging Innovations in AMR diagnostics and therapeutics.

Category	Innovation	Description	Status
Diagnostics	Rapid Molecular Diagnostics	PCR, multiplex assays for quick pathogen & resistance gene identification	In clinical use
	Next‐Generation Sequencing (NGS)	Whole‐genome sequencing for comprehensive resistance profiling	Emerging
	CRISPR‐Based Diagnostics	Use of CRISPR‐Cas systems for targeted detection of resistance genes	Experimental
	Point‐of‐Care (POC) Testing	Handheld or bedside tests for AMR detection	Expanding adoption
	AI‐Driven Diagnostic Algorithms	AI models analyzing EHR & lab data to predict resistance patterns	Pilot studies
Therapeutics	Phage Therapy	Use of bacteriophages to target resistant bacteria	Compassionate use/early trials
	Monoclonal Antibodies	Antibodies targeting bacterial toxins or virulence factors	Clinical trials
	Antimicrobial Peptides	Synthetic or natural peptides with broad‐spectrum antimicrobial activity	Preclinical/early trials
	CRISPR‐Based Antimicrobials	Gene‐editing tools to selectively kill resistant bacteria	Preclinical
	Nanotechnology‐Based Drug Delivery	Nanocarriers to improve antibiotic delivery and reduce resistance	Emerging
	Antibiotic Adjuvants	Compounds that restore efficacy of existing antibiotics	Clinical trials
	Microbiome Restoration Therapies	Fecal microbiota transplantation and probiotics	Selective use/trials
Stewardship tools	Clinical Decision Support Systems (CDSS)	Software integrated with EHRs to guide appropriate antibiotic prescribing	Widely implemented
	Surveillance and Big Data Analytics	AMR trend monitoring using real‐time data	In use/expanding

Several studies have demonstrated that the fusidic acid resistance effect in *S. aureus* and *S. enterica* (acquired by amino acid exchanges within the elongation factor G, EF‐G) is linked to a substantial decrease in bacterial fitness [127, 137, 138]. This is relevant to intragenic mutations [137]. Many times, particular changes to the fusA gene can significantly lessen these harmful effects [127]. One crucial feature of compensating mutations is that, occasionally, other resistant mutations result in compensation [139]. In the instance of *P. aeruginosa* rifampicin‐resistant mutations, this epistatic influence was noted. Resistance mutations in various genes may potentially enable compensatory fitness costs in addition to intragenic compensatory mutation. This is an example of secondary mutations in topoisomerase IV (a target of quinolones) genes, which can offset the expense of primary mutations in marR [138].

To reduce fitness costs, the abuse of antibiotics should be regulated. To stop the abuse, misuse, and eventual growth in AMR of antibiotics, it is crucial to strike an equilibrium between autonomy and the significance of antimicrobial stewardship. Programs that encourage the responsible use of antibiotics, or “antimicrobial stewardship,” can help to keep this equilibrium (Table 3). Healthcare professionals can promote safe usage of antibiotics through the use of such initiatives without interfering with people's right to privacy. These programs involve collaborative decision‐making among patients and medical professionals, ensuring only essential prescriptions are written for antibiotics, and tracking the consumption of antibiotics to spot and treat abuse trends [140].

## 9 Genomic and Metagenomic Approaches to Study Antibiotic Resistance Evolution

A mix of traditional culture‐dependent phenotypic techniques (such as broth microdilution, disk diffusion, gradient diffusion, and automated systems) and culture‐independent fast molecular techniques is currently used by the majority of clinical microbiology labs to forecast antibiotic resistance [141]. This method offers quicker turnaround times employing molecular approaches for a portion of the intended pathogens while maintaining historical results data and the species range of traditional screening [141]. It is important to note that this strategy is not ideal. For instance, only a small portion of important resistance indicators from quite a few clinical pathogens are amenable to fast molecular analysis. Any culture‐dependent approach has drawbacks, including the need for in vitro growth, longer turnaround periods for slow‐growing microorganisms, bias against dominating microbial populations, and the possibility of contaminant overgrowth [141].

However, the adoption of genomic‐based resistance predictions is a little delayed in the discipline of clinical microbiology. Turnaround time and additional expenditures as comparison to conventional procedures, as well as the dearth of conclusive outcomes data indicating clinical efficacy, are major issues of dispute. Studies have revealed that certain elements might not be as problematic as once thought [142]. Another important issue is the dearth of reliable predictive techniques, particularly for Gram‐negative bacteria like *P. aeruginosa*. There are benefits to using genomic‐based resistance identification in a clinical microbiology lab. First, the turnaround time for infections with slow growth rates could be accelerated. Second, a larger variety of microorganisms may be tested in labs. Fastidious bacteria and other microorganisms, such as fungi or parasites, may fall under this category. Third, an isolated relationship could be assessed by a clinical laboratory. It is crucial for laboratories to detect contamination, evaluate unsuccessful therapy versus new infections, as well as infection management and preventive procedures. Instead of submitting an isolate, such isolate‐relatedness data might be given straight to public health, which might result in a quicker identification of epidemics [143, 144].

An alternate method involves doing Metagenomics Next Generation Sequencing directly from a clinical specimen, capturing all of the pathogenic nucleic acids, other microorganism nucleic acids, and the individual's human nucleic acids, as opposed to sequencing pure cultures of a separate microbe [145] (Table 3). Avoiding culture bottlenecks is a key benefit of this objective method of identifying pathogens, which is why clinical metagenomic sequencing is becoming more popular [146, 147]. This strategy can be very beneficial for infections that develop slowly, like Mycobacterium species [148]. According to a prospective examination of “cerebrospinal fluid for metagenomic sequencing,” 22% (13/58) of infections may be detected through sequencing rather than by conventional laboratory techniques [147].

Additionally, metagenomic sequencing gave clinicians other advantages, such as the detection of *Klebsiella aerogenes* (formerly known as *Enterobacter aerogenes*) ARGs and the prediction of HIV‐1 treatment resistance [147]. The disadvantage of this approach is the fact that there will be no isolate available for phenotypic susceptibility testing, which would impede confirmation of ARG‐based resistance predictions (Table 3). Additionally, clinical evaluation of the results can be difficult when numerous putative infections have been identified with unique readings [149].

## 10 Recommendations

### 10.1 Development of New Antibiotics and Combination Therapies

**Figure 4:**
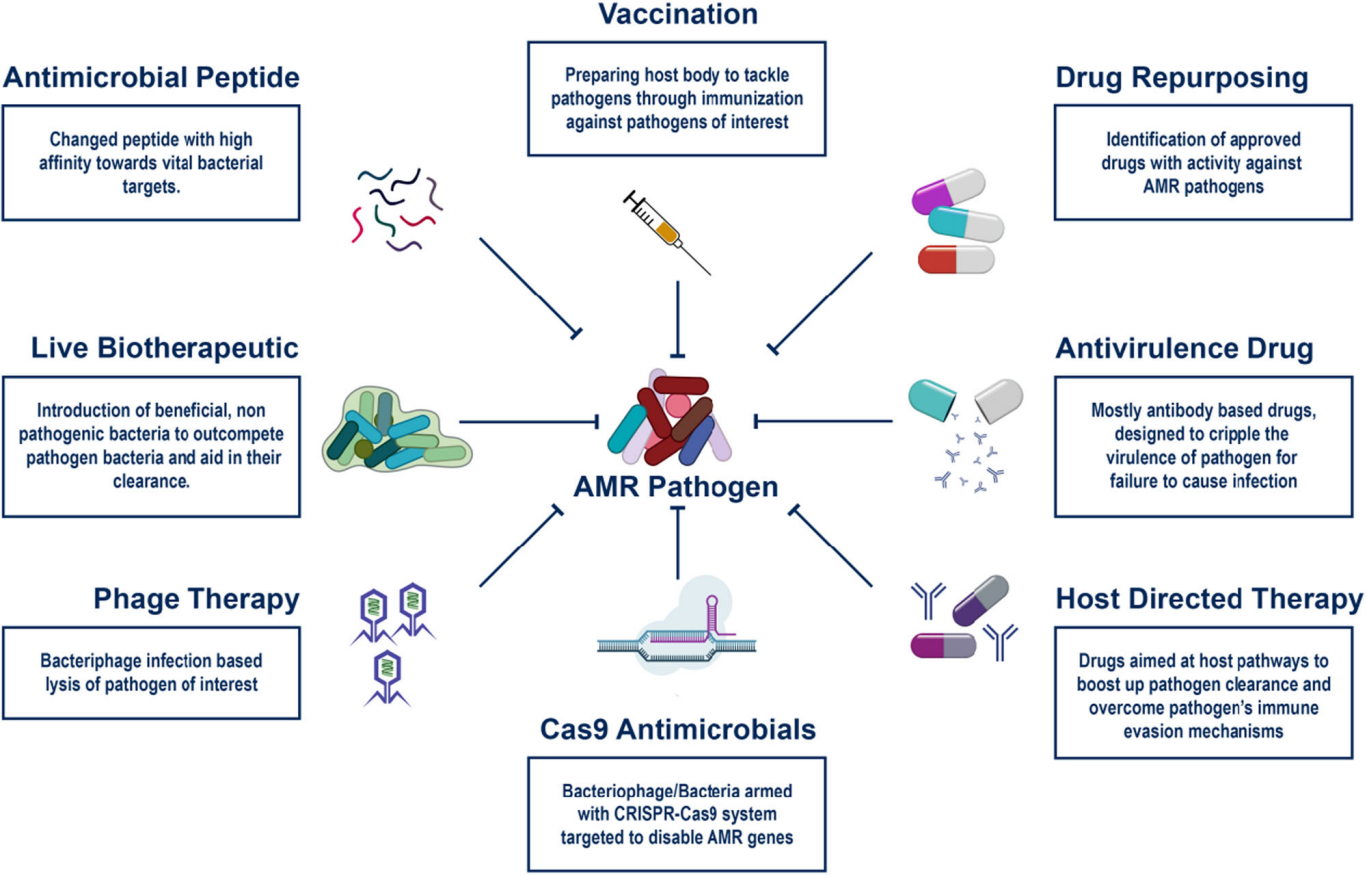
Development of new antibiotics and combination therapies. Development of new antibiotics and combination therapies involves innovative strategies to overcome antimicrobial resistance. This includes designing novel antibiotic classes, modifying existing drugs to enhance efficacy, and utilizing combination therapies that target multiple resistance mechanisms simultaneously. Approaches such as β‐lactamase inhibitors, synergistic drug combinations, and bacteriophage therapy aim to restore antibiotic potency and reduce resistance development.

To overcome emerging antibiotic resistance mechanisms, several strategies can be implemented. Developing new antibiotics with novel mechanisms of action that target bacterial processes not affected by existing drugs is crucial. Utilizing advanced technologies such as high‐throughput screening, genomics, and bioinformatics can help identify new antibiotic candidates, including natural products, synthetic compounds, and biologics. Additionally, focusing on nontraditional targets like bacterial virulence factors, biofilm formation, and quorum sensing can weaken bacteria and enhance the effectiveness of existing antibiotics [150] (Figure 4).

Using synergistic combinations of existing antibiotics can enhance efficacy and reduce the likelihood of resistance development by attacking bacteria through multiple mechanisms simultaneously. Pairing antibiotics with adjuvants that inhibit resistance mechanisms, such as beta‐lactamase inhibitors, or with nonantibiotic agents like immunomodulators or efflux pump inhibitors, can further improve outcomes. Investing in rapid diagnostic tools to quickly identify bacterial infections and their resistance profiles allows for targeted therapy with the most effective antibiotics, reducing misuse and slowing resistance spread [151].

### 10.2 Targeting Nontraditional Bacterial Vulnerabilities to Overcome Resistance

Overcoming emerging antibiotic resistance necessitates innovative strategies that target nontraditional bacterial vulnerabilities. One approach involves targeting bacterial communication pathways, known as quorum sensing, by using inhibitors or autoinducer analogs to disrupt the coordination necessary for virulence factor production and biofilm formation. Another strategy focuses on disrupting biofilm formation with dispersal agents like dispersin B or DNase and applying anti‐biofilm coatings on medical devices to prevent infection [152].

Enhancing the host immune response through immunomodulatory therapies or vaccines can also reduce the reliance on antibiotics. Targeting bacterial metabolism with pathway inhibitors or nutrient limitation strategies can starve bacteria or inhibit their growth. Disrupting bacterial stress responses with heat shock protein inhibitors or stress response modulators can impair bacterial survival under stress conditions. Targeting bacterial membranes with disruptors like AMPs or synthetic polymers and inhibiting essential outer membrane proteins can compromise bacterial survival. Combination therapies, including synergistic drug combinations and adjunctive therapies, can enhance treatment outcomes and reduce the development of resistance. These strategies represent a multi‐faceted approach to combat antibiotic resistance by exploiting bacterial vulnerabilities beyond traditional antibiotics [153].

### 10.3 Immunotherapies and Phage Therapy as Alternative Approaches

Emerging antibiotic resistance poses a significant challenge to public health, necessitating the development of alternative approaches like immunotherapies and phage therapy. Immunotherapies involve several strategies. Monoclonal antibodies can target specific bacterial components, neutralizing the pathogen without relying on traditional antibiotics, or enhance the immune response by tagging bacteria for destruction by immune cells. Vaccines, both preventative and therapeutic, can help prevent infections or boost the immune response in infected individuals. Immune modulators, such as cytokine therapy, can enhance the immune system's ability to fight resistant bacteria, and immune checkpoint inhibitors might be explored for chronic bacterial infections.

Phage therapy, which utilizes bacteriophages to target and lyse specific bacterial strains, offers a promising alternative, especially against MDR organisms. However, both phage therapy and immunotherapies face critical limitations. Regulatory challenges remain significant, as phages are biologics with high variability and require strain‐specific approval pathways that are not yet standardized across jurisdictions. Moreover, the human immune system may neutralize administered phages, reducing efficacy or triggering inflammatory responses. Immunotherapies, while highly targeted, can also carry the risk of off‐target effects, immune‐related adverse events, and high production costs. The need for individualized treatment design further complicates large‐scale implementation.

Combining these strategies with personalized medicine approaches can further improve outcomes. Rapid diagnostic tools can identify specific bacterial pathogens and their resistance profiles, allowing for personalized treatment plans. Research and development efforts are crucial to understanding resistance mechanisms and developing more effective treatments. Clinical trials are needed to establish the safety and efficacy of these alternative therapies, ensuring they can be integrated into standard medical practice. Implementing these strategies provides a robust defense against antibiotic‐resistant infections, leveraging the specificity and adaptability of immunotherapies and phage therapy to overcome the limitations of traditional antibiotics [154, 155, 156].

### 10.4 Limitations of Current Approaches to Combat AMR

The escalating threat of AMR demands urgent and coordinated global intervention. Current strategies to combat AMR include antibiotic stewardship programs, infection prevention measures, surveillance systems, novel drug development, and alternative therapies such as bacteriophage therapy, AMPs, and CRISPR‐based gene editing. However, these approaches face significant challenges that limit their effectiveness.

One of the most critical limitations is the slow pace of new antibiotic discovery. Despite increasing resistance, the development pipeline for novel antibiotics remains insufficient, largely due to the high cost, lengthy approval processes, and low financial incentives for pharmaceutical companies. The emergence of MDR and XDR bacterial strains has outpaced drug development, leaving clinicians with limited treatment options.

Antibiotic stewardship programs, while effective in promoting the judicious use of existing antibiotics, are often inconsistently implemented, particularly in low‐resource settings. Many healthcare systems lack the infrastructure, trained personnel, and regulatory frameworks to enforce antibiotic prescribing guidelines. Additionally, the misuse and overuse of antibiotics in human medicine, agriculture, and animal husbandry continue to drive resistance despite global awareness efforts. The widespread availability of over‐the‐counter antibiotics in certain regions exacerbates the problem, contributing to the selection of resistant bacterial strains. Surveillance and monitoring systems for AMR are crucial for tracking resistance patterns and informing policy decisions. However, these systems are often fragmented, underfunded, and inadequate in many parts of the world. Low‐income countries, in particular, face difficulties in establishing robust AMR surveillance due to limited laboratory capacity, lack of standardized protocols, and insufficient data‐sharing mechanisms. Without comprehensive global data, efforts to contain AMR remain suboptimal. Alternative therapies, such as bacteriophage therapy and AMPs, offer promising solutions but are still in experimental stages. Phage therapy, for instance, faces challenges related to regulatory approval, strain specificity, and potential immune responses. Similarly, AMPs, while effective against resistant pathogens, have issues related to stability, toxicity, and large‐scale production. CRISPR‐based approaches for targeting resistance genes are in early development, requiring further research before clinical application.

Furthermore, the rapid evolution of resistance mechanisms among microbes presents an ongoing challenge. Bacteria have demonstrated remarkable adaptability, acquiring resistance through HGT, efflux pumps, and enzymatic degradation of antibiotics. The ability of pathogens to develop resistance faster than the rate of new drug discovery underscores the urgency of innovative solutions.

Addressing these limitations requires a multifaceted and globally coordinated approach. Strengthening antibiotic stewardship programs, increasing investment in research and development, enhancing global surveillance networks, and implementing stringent regulations on antibiotic use in agriculture are critical steps. Additionally, fostering public‐private partnerships, encouraging novel financing models for antibiotic development, and promoting interdisciplinary collaboration between microbiologists, clinicians, and policymakers will be essential in the fight against AMR. Without decisive action, AMR threatens to undermine modern medicine, rendering common infections untreatable and routine surgeries high‐risk procedures.

### 10.5 Limitations of This Review

This review is subject to several inherent limitations. The inclusion of only English‐language, peer‐reviewed publications may introduce language and publication bias. Reliance on major databases (e.g., PubMed, Scopus, ScienceDirect) may exclude regional or gray literature, potentially omitting important data from underrepresented regions. Furthermore, variations in surveillance standards and reporting methodologies across countries may limit the generalizability of findings. These constraints underscore the need for more inclusive and standardized global research efforts.

## 11 Conclusion

The continuous evolution of AMR underscores the urgent need to understand the intricate relationship between microorganisms and antibiotics, as it remains one of the most significant global health challenges. Addressing AMR in contemporary healthcare requires a comprehensive approach to elucidate the mechanisms through which microorganisms adapt and evolve to withstand antibiotic treatment. To combat AMR and preserve the effectiveness of antimicrobial therapies, a multifaceted strategy is essential. This includes advancing scientific research to develop novel antibiotics and alternative therapies, strengthening antimicrobial stewardship programs, and enhancing global surveillance efforts. However, scientific advancements alone are insufficient. Success in mitigating AMR relies on coordinated action and collaboration among healthcare professionals, researchers, policymakers, and the pharmaceutical industry.

Healthcare providers must implement evidence‐based prescribing practices, while researchers should continue exploring innovative treatment strategies and rapid diagnostic tools. Policymakers play a crucial role in enforcing regulations to curb antibiotic misuse in healthcare and agriculture, and pharmaceutical companies must prioritize the development of new antimicrobial agents despite financial and regulatory challenges. Furthermore, international partnerships and funding initiatives are necessary to support low‐ and middle‐income countries in their fight against AMR.

The battle against AMR requires immediate, sustained, and collective action. By fostering interdisciplinary collaboration, promoting responsible antibiotic use, and investing in groundbreaking research, we can work toward a future where the threat of AMR is effectively controlled. If we act now, we have the opportunity to preserve the efficacy of antimicrobial therapies and safeguard global public health for generations to come.

## Author Contributions

**Abubakar Nazir:** conceptualization, writing – original draft, writing – review and editing, project administration, supervision, validation, visualization. **Awais Nazir:** writing – original draft, writing – review and editing. **Varisha Zuhair:** writing – original draft, writing – review and editing. **Shafaq Aman:** writing – review and editing, formal analysis, supervision. **Safi Ur Rehman Sadiq:** conceptualization, project administration, writing – review and editing, validation, supervision. **Abdul Haseeb Hasan:** writing – original draft, writing – review and editing. **Maryam Tariq:** writing – original draft, writing – review and editing. **Latif Ur Rehman:** writing – original draft, writing – review and editing. **Mubarak Jolayemi Mustapha:** writing – original draft, writing – review and editing. **Deusdedith Boniphace Bulimbe:** writing – original draft, writing – review and editing.

## Ethics Statement

The authors have nothing to report.

## Conflicts of Interest

The authors declare no conflicts of interest.

## Transparency Statement

The lead author, Abubakar Nazir, affirms that this manuscript is an honest, accurate, and transparent account of the study being reported; that no important aspects of the study have been omitted; and that any discrepancies from the study as planned (and, if relevant, registered) have been explained.

## Data Availability

Data sharing is not applicable to this article as no data sets were generated or analyzed during the current study.
